# The Predictive Effect of “Real Amylase Value”: A More Accurate Predictor for Postoperative Pancreatic Fistula

**DOI:** 10.1111/ans.70276

**Published:** 2025-08-07

**Authors:** Ahmet Cihangir Emral, Gökay Çetinkaya, Kürşat Dikmen, Mustafa Kerem

**Affiliations:** ^1^ Department of General Surgery Atılım University Faculty of Medicine Ankara Turkey; ^2^ Department of General Surgery Sağlık Bilimleri University Gülhane Eğitim Ve Araştırma Hastanesi Ankara Turkey; ^3^ Department of General Surgery Gazi University Faculty of Medicine Ankara Turkey

**Keywords:** amount of drain amylase, drain amylase concentration, pancreatic fistula, real amylase value

## Abstract

**Background:**

Postoperative pancreatic fistula (POPF) is a common and serious complication following pancreatic surgery. While several studies have attempted to predict the development of POPF using drain amylase concentration, predictive values vary widely due to factors like abdominal irrigation and chylous drainage, which can dilute the amylase levels. This study aims to evaluate whether the “Real Amylase Value” (RAV), calculated as the product of drain amylase concentration and drainage volume, provides a more reliable prediction of POPF compared to conventional amylase concentration. Better prediction of pancreatic fistula development could lead to closer clinical monitoring of these patients, reassessment of hospital stay duration, and more careful management of drains over an extended period. Additionally, carefully managing the timing of drain removal may improve patient recovery and discharge process.

**Methodology:**

Data from 198 patients who underwent pancreaticoduodenectomy (PD) and distal pancreatectomy (DP) were retrospectively analyzed. Drain amylase concentrations and drainage volumes were measured on postoperative days (POD) 1 and 3, and the RAV (U) was calculated. Real Amylase Value (RAV) (U) was calculated using the formula: RAV (U) = Drain Amylase Concentration (U/L) × Drainage Amount (L). Predictive values for POPF were evaluated using receiver operating characteristic (ROC) curve analysis, comparing conventional amylase concentration (U/L) and RAV (U).

**Results:**

On POD1, the RAV (U) demonstrated greater predictive value for POPF compared to the conventional drain amylase concentration (U/L) with an area under the ROC curve (AUROC) of 0.85 versus 0.79, respectively. Similarly, on POD3, RAV showed superior predictive accuracy (AUROC 0.89) compared to amylase concentration (AUROC 0.79).

**Conclusion:**

The RAV (U) offers a more accurate and reliable prediction of POPF than traditional drain amylase concentration (U/L), with improved sensitivity and specificity. This method could refine clinical management, particularly in the timing of drain removal and early intervention strategies for patients at high risk of developing pancreatic fistulas.

## Graphical abstract

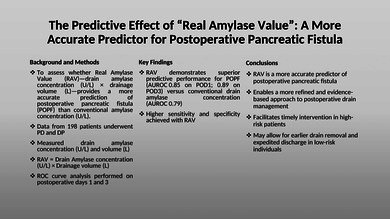

## 1 Introduction

Despite advancements in surgical techniques and perioperative care, pancreatic surgery continues to have a mortality rate of approximately 5% and a morbidity rate of 30%–50% [1, 2]. Postoperative pancreatic fistula (POPF) is a common and significant complication following pancreatic surgery [1, 3]. The incidence of POPF following pancreaticoduodenectomy (PD) ranges from 5% to 30%, while it ranges from 5% to 40% after distal pancreatectomy (DP) [4, 5]. Complications associated with POPF include intra‐abdominal abscesses, delayed gastric emptying, bleeding, sepsis, and death. Early diagnosis of POPF allows for timely intervention (such as adjusting antibiotics or initiating parenteral nutrition) before clinical deterioration occurs. Additionally, the recovery process can be expedited with early enteral nutrition in patients who do not develop POPF [4, 6]. Furthermore, early drain removal (postoperative day [POD] < 4) has been shown to reduce the incidence of complications compared to later drain removal (POD > 5) [6]. Drains are typically removed at the surgeon's discretion after the risk of POPF has been excluded. Predicting the development of POPF and managing the timing of drain removal may enhance patient recovery and discharge [6].

Several studies have sought to predict the development of POPF, with efforts focused on determining predictive values based on drain amylase concentration. However, different centers have reported varying predictive values [4, 6]. In these studies, drain amylase concentrations measured in relation to POPF development are expressed as “Unit (U)/Liter (L).” However, factors such as saline irrigation of the abdomen, intraoperative bleeding, ascites, and chylous drainage following extensive lymphatic dissection may dilute the drain amylase concentration, potentially leading to inaccurate predictive values.

To date, no study has calculated the number of units (U) of pancreatic amylase leakage or determined a predictive value based on this measurement. Moreover, whether pancreatic amylase concentration or the amount of pancreatic amylase provides a more accurate prediction of POPF has not been explored. The aim of this study is to investigate whether examining pancreatic amylase as a unit, rather than the conventional pancreatic drain amylase concentration (unit/L), offers a more meaningful predictive value in terms of both sensitivity and specificity. If the unit quantity of pancreatic amylase proves to be a more accurate predictive tool, it could allow for more precise predictions regarding the development of pancreatic fistulas. This, in turn, could lead to closer clinical monitoring of these patients, a reassessment of hospital stay duration, and more careful management of drains over an extended period.

## 2 Methods

Patient information was obtained from prospectively completed standardized daily follow‐up records. Data from 210 patients prospectively recorded in our pancreatic surgery database between January 1, 2020, and July 1, 2024, were retrospectively evaluated. Ethical approval for this study was granted by the Gazi University Faculty of Medicine Ethics Committee (No. 09‐2021). Patients considered unresectable intra‐operatively or those who underwent total pancreatectomy were excluded from the study. The data of 198 patients who underwent resectable pancreaticoduodenectomy (PD) and distal pancreatectomy (DP) were included in the analysis.

In 2016, the International Study Group of Pancreatic Fistula (ISGPF) updated the definition of pancreatic fistula. While POPFs were previously classified into Grade A, Grade B, and Grade C, the latest update redefined Grade A as a biochemical leak and excluded it from the pancreatic fistula category. Specifically, Grade A was defined as a drain amylase concentration greater than three times the normal serum amylase value on the third postoperative day, without any clinical findings. Grade B was characterized by the presence of drainage lasting more than 3 weeks, without signs of organ failure or infection related to POPF, but with mild signs of infection (such as leukocytosis and mild fever) requiring antibiotic treatment, clinical changes in POPF management, percutaneous or endoscopic drainage, repositioning of the operatively placed drains, or angiographic procedures for bleeding. Grade C included re‐operation, organ failure, or mortality [7]. Fistula classifications were made according to ISGPF criteria and recorded in our database. Only Grade B and C fistulas were considered pancreatic fistulas; Grade A was regarded as a biochemical leak and excluded from the analysis.

All surgeries were performed by the same standardized surgical team. A 10 mm flat silicone drain was placed in the pancreatectomy area for patients undergoing DP. For patients undergoing pancreaticoduodenectomy, a 10 mm silicone flat drain was placed from the foramen of Winslow to beneath the pancreaticojejunostomy. Octreotide was not routinely used in the postoperative management of pancreatic resections in our institution.

In our database, postoperative day 1 was defined as 24 h after the completion of surgery. The amount of drainage and drain amylase values (U/L) for each patient were recorded on the first and third days, based on each 24‐h period after the patient arrived in the recovery unit. Based on our experience in pancreatic surgery, and in the absence of any additional risk factors (such as diabetes, a history of neoadjuvant therapy, immunosuppression, or a soft pancreas), drains with an amylase concentration below our institution's cutoff value and serous drainage are removed on the third postoperative day. Patients with pancreatic drain amylase levels (unit/L) above the clinic's predictive value were considered to have a biochemical leak (Grade A fistula); however, no changes were made to treatment unless clinical symptoms warranted it. In cases where the drain volume decreased or stopped suddenly during the postoperative period, the drain was flushed with 10 cc of sterile saline to rule out obstruction. For patients with clinical suspicion of complications, abdominal ultrasonography or tomography was used to assess for intra‐abdominal collections. For drains that were not removed, patients were monitored for vital signs, drain amylase levels, drain volume, and drain color on postoperative days 5, 7, 14, and 21, with drainage management adjusted accordingly. These patients received closer clinical follow‐up. All patients predicted to develop pancreatic fistula were evaluated with abdominal CT on the 10th postoperative day, and drainage was performed when necessary.

Real Amylase Value (RAV) (U) was calculated using the formula: RAV (U) = Drain Amylase Concentration (U/L) × Drainage Amount (L).

### 2.1 Statistical Analysis

All data were transferred to a computer environment, and statistical analysis was performed using SPSS 20.0 software (SPSS Inc., Chicago, IL, USA). A *p*‐value of < 0.05 was considered statistically significant. Independent‐samples *t*‐tests were used to compare the means of one variable between two groups, while paired‐samples *t*‐tests were used to compare the means of two variables within a single group. For ROC analysis, the development of Grade B and C pancreatic fistulas was defined as the primary endpoint. Areas under the receiver operating characteristic (ROC) curve were calculated to evaluate amylase concentration (U/L) and RAV (U) as predictors of POPF. ROC analysis was conducted based on patients' drain amylase (U/L) and RAV (U) values on the first and third days. The results of the ROC analysis, including sensitivity and 1‐specificity values, were examined, and the drain amylase (U/L) and RAV (U) values with the highest sensitivity and specificity were selected as cut‐off points. Finally, the Area Under the Curve (AUC) and *p*‐values were compared.

## 3 Results

Data from a total of 198 patients (88 women, 110 men) were analyzed. The mean age of the patients was 60.2 ± 11.2 years (median 62 years, range 16–85 years). The median time for drain removal in all patients was 3 days (min. 2‐max. 28). A total of 43 patients developed pancreatic fistulas (grade B/C). Among these patients, 4 required surgical intervention via laparotomy, and 15 required interventional radiology procedures, totaling 19 patients who needed postoperative drainage due to pancreatic fistula. Information regarding the patients' surgeries and the development of pancreatic fistulas is provided in Table 1.

**TABLE 1:** Types of operations performed and the incidence of pancreatic fistula in patients.

Operations performed	Number of patients (%)	Number of patients developing pancreatic fistula (%)
Laparoscopic DP	18 (9.1)	3 (16.6)
DP	27 (13.6)	10 (37)
PD	153 (77.3)	30 (19.6)
Total operation	198 (100)	43 (21.7)

**FIGURE 1:**
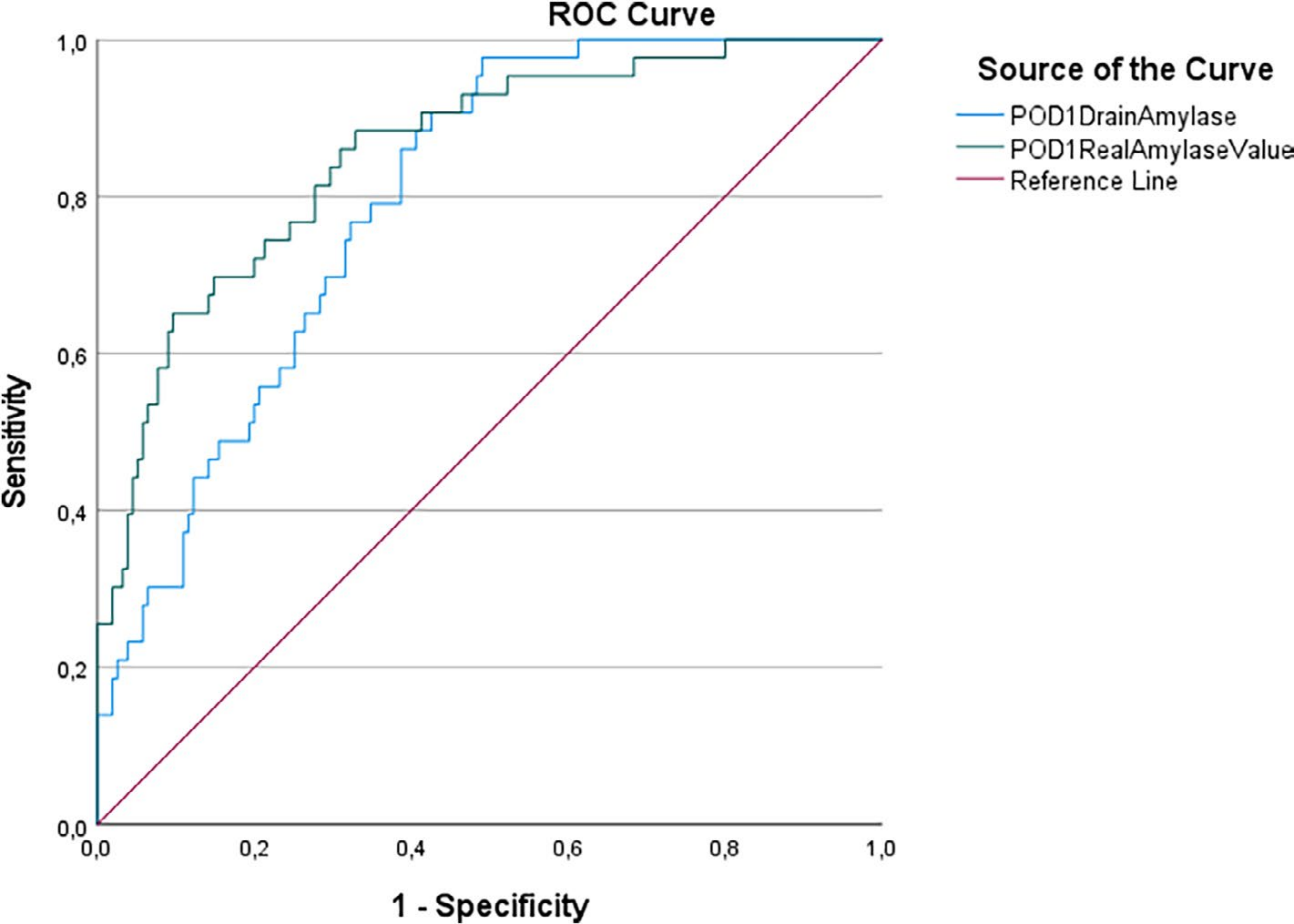
Comparison of drain amylase concentration (U/L) and real amylase value (RAV) (U) on postoperative day 1 (POD1) using ROC curve analysis to predict pancreatic fistula development in patients.

When evaluating the conventional drain amylase concentration (U/L) and the Real Amylase Value (RAV) (U) using ROC curve analysis on postoperative day 1 (POD1), the RAV value demonstrated statistically greater significance than the conventional drain amylase concentration in predicting the development of pancreatic fistula (*p* < 0.01). In this study, the predictive values were as follows: for POD1 Amylase (U/L) at 1060 U/L, we obtained 69.8% sensitivity and 71% specificity, while for POD1 RAV (U) at 211 U, sensitivity was 72.1% and specificity was 80%. The area under the receiver operating characteristic (AUROC) curve was 79.5% for POD1 Drain Amylase (U/L) and 85.4% for POD1 RAV. The ROC curve analysis results for POD1 are shown in Figure 1.

**FIGURE 2:**
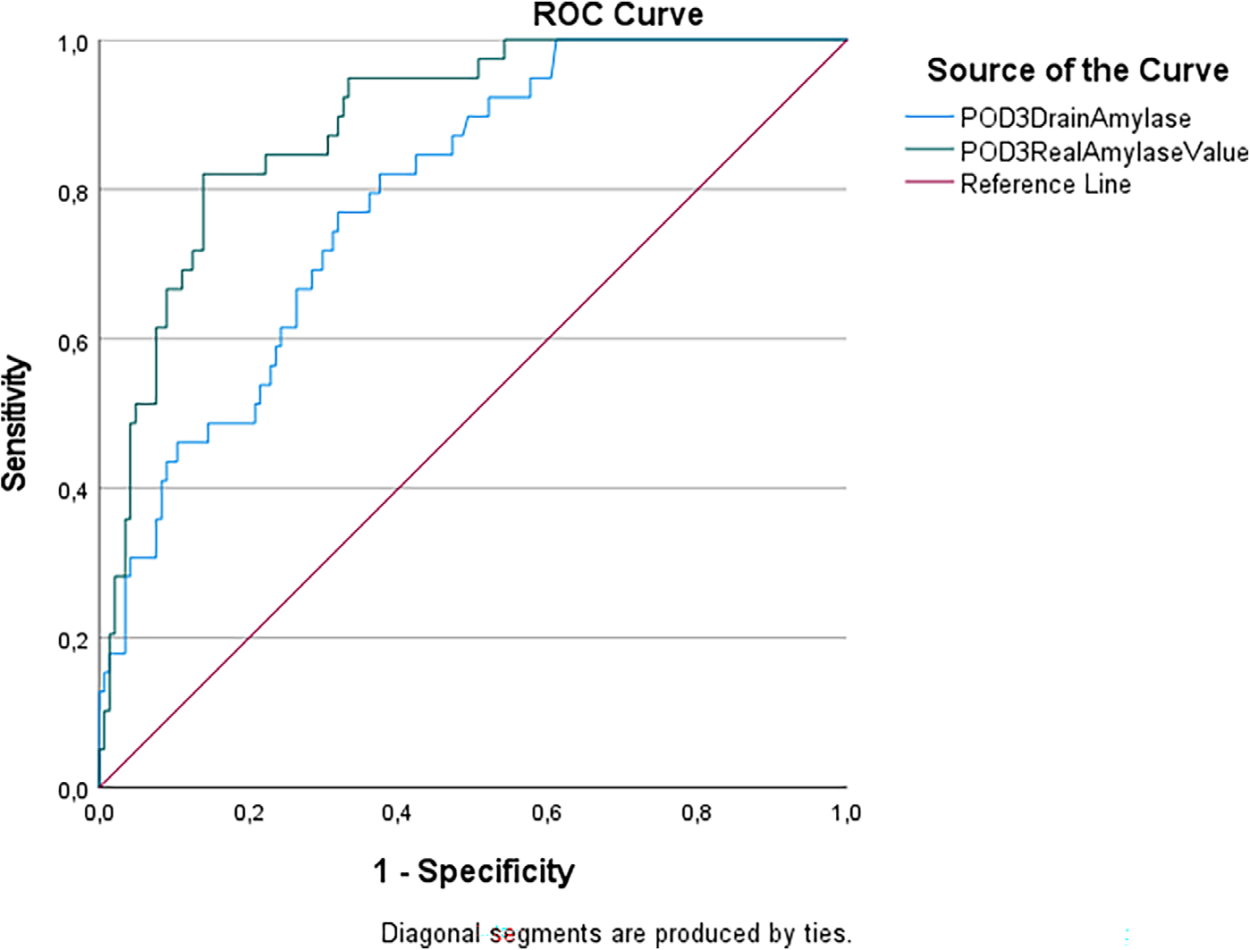
Comparison graphic of drain amylase concentration (U/L) and RAV (U) at postoperative day 3 (POD3) with ROC curve analysis to predict pancreatic fistula development of patients.

When we evaluated the conventional drain amylase concentration (U/L) and the Real Amylase Value (RAV) (U) using ROC curve analysis on postoperative day 3 (POD3), the RAV value again demonstrated greater statistical significance than the conventional drain amylase concentration in predicting the development of pancreatic fistula (*p* < 0.01). In this analysis, we found the following predictive values: for POD3 Amylase (U/L) at 241 U/L, sensitivity was 79.1% and specificity was 68.1%, while for POD3 RAV (U) at 40 U, sensitivity was 83.7% and specificity was 86.1%. The AUROC for POD3 Drain Amylase (U/L) was 79.7%, and for POD3 RAV (U), it was 89.6%. The ROC curve analysis results for POD3 are presented in Figure 2.

## 4 Discussion

Pancreatic leakage is one of the major complications frequently encountered after pancreaticoduodenectomy (PD) and distal pancreatectomy (DP). Several factors, such as soft pancreatic tissue, pancreatic duct diameter, long operation time, intraoperative blood loss, blood transfusions, and low surgical volume centers, have been identified as risk factors for pancreatic fistula (PF) development [6, 8, 9, 10]. Due to the multifactorial nature of postoperative pancreatic fistula (POPF) development and the evolving approaches to postoperative patient follow‐up, no perfect method has yet been established to predict the occurrence of POPF. Numerous studies have attempted to determine predictive drain amylase concentration (U/L) values as cut‐off points to detect early POPF development. These studies report varying levels of sensitivity and specificity based on their respective cut‐off values [4, 5, 6].

While centers that adopt lower cut‐off values may result in excessive treatment, considering the potential for POPF development, Lee et al. [11] demonstrated that a low drain amylase concentration suggests that a pancreatic fistula is unlikely to occur. In their study, using a cut‐off value of < 90 U/L, they achieved 96.7% sensitivity and 36.5% specificity. Despite the low cut‐off indicating that a pancreatic fistula is unlikely, values above this threshold may still suggest the potential for fistula development, which could lead to prolonged drain retention and delayed removal. Consequently, failure to remove the drain early may increase the incidence of complications [12, 13]. Additionally, factors such as saline irrigation of the abdomen, intraoperative bleeding, ascites, and chylous drainage following extensive lymphatic dissection can dilute the drain amylase concentration, resulting in false predictive values.

Conversely, centers with higher cut‐off values tend to better identify the development of POPF, but many patients whose values fall below the cut‐off may not receive an early diagnosis of POPF. Kawai et al. [14], in their study, found that when the drain amylase concentration cut‐off was set at > 4000 U/L, sensitivity was 62.2% and specificity was 89%. The updated definition of grade A fistulas (biochemical leak) by the ISGPF in 2016, which is based on high drain amylase concentrations that do not affect the clinical course, underscores the importance of drain volume in our opinion. The fact that some patients with high drain amylase concentrations did not develop pancreatic fistulas may be related to the drainage volume. Zhou et al. [15] identified postoperative drainage volume on day 1 after distal pancreatectomy as a significant (*p* = 0.03) independent risk factor for pancreatic fistula development. This finding highlights the importance of both drain volume and amylase concentration in assessing the risk of POPF.

According to the results of this study, the Real Amylase Value (RAV) (U) yielded statistically more significant findings and was more valuable in terms of both sensitivity and specificity compared to conventional drain amylase concentration. Zelga et al. [6, 16], in their study, found that using a predictive cut‐off value of > 1400 U/L for POD1 drain amylase concentration yielded 76% sensitivity and 70% specificity. Maggio et al. [17], using a cut‐off of 2000 U/L on POD1, reported 74.3% sensitivity and 62.1% specificity. Both studies found relatively low specificity, which can be attributed to measuring amylase as a concentration. In contrast, in this study, when we selected 1060 U/L as the cut‐off for POD1 drain amylase concentration, we achieved 69.2% sensitivity and 70.1% specificity. On the other hand, when we evaluated POD1 and POD3 RAV (POD1 RAV: 211 U, 72.1% sensitivity, 80% specificity; POD3 RAV: 40 U, 83.7% sensitivity, 86.1% specificity), we found statistically more significant results in terms of both sensitivity and specificity.

Hepatobiliary surgeons have observed that some patients with high drain amylase concentrations (classified as biochemical leaks according to the ISGPF) do not develop fistulas, while others with low drain amylase concentrations may still develop pancreatic fistulas. As this study demonstrates, evaluating drain amylase as a unit rather than as a concentration provides surgeons with better insight into the likelihood of pancreatic fistula development.

The main limitation of this study is its retrospective design, as well as the relatively small number of patients who developed pancreatic fistulas. Further prospective studies with larger sample sizes are needed to validate these findings and to refine predictive models for POPF.

## 5 Conclusion

This study provides a foundation for future research and may offer valuable insights into drainage management for patients with very high drain amylase concentrations (e.g., 10 000 U/L) but minimal drainage (e.g., 10 cc), or those with very low drain amylase concentrations (e.g., 100 U/L) but substantial drainage (e.g., 1000 cc). Additional studies are necessary to further validate and strengthen the findings of this research.

## Author Contributions

**Ahmet Cihangir Emral:** formal analysis, investigation, methodology, writing – original draft. **Gökay Çetinkaya:** formal analysis, investigation, methodology, supervision. **Kürşat Dikmen:** data curation, formal analysis, investigation, methodology, supervision. **Mustafa Kerem:** conceptualization, data curation, formal analysis, investigation, methodology, project administration, supervision.

## Conflicts of Interest

The authors declare no conflicts of interest.

## Data Availability

Data sharing is not applicable to this article as no new data were created or analyzed in this study.
